# A Fully Integrated and Miniaturized Heavy-metal-detection Sensor Based on Micro-patterned Reduced Graphene Oxide

**DOI:** 10.1038/srep33125

**Published:** 2016-09-12

**Authors:** Xing Xuan, Md. Faruk Hossain, Jae Yeong Park

**Affiliations:** 1 Department of Electronic Engineering, Micro/Nano Devices & packaging Lab., Kwangwoon University, 447-1, Wolgye-Dong, Nowon Gu, Seoul, 139-701, Korea

## Abstract

For this paper, a fully integrated and highly miniaturized electrochemical sensor was designed and fabricated on a silicon substrate. A solvothermal-assisted reduced graphene oxide named “TRGO” was then successfully micro-patterned using a lithography technique, followed by the electrodeposition of bismuth (Bi) on the surface of the micro-patterned TRGO for the electrochemical detection of heavy metal ions. The fully integrated electrochemical micro-sensor was then measured and evaluated for the detection of cadmium and lead-heavy metal ions in an acetic-acid buffered solution using the square wave anodic stripping voltammetry (SWASV) technique. The fabricated micro-sensor exhibited a linear detection range of 1.0 μg L^−1^ to 120.0 μg L^−1^ for both of the metal ions, and detection limits of 0.4 μg L^−1^ and 1.0 μg L^−1^ were recorded for the lead and cadmium (S/N = 3), respectively. Drinking-water samples were used for the practical assessment of the fabricated micro-sensor, and it showed an acceptable detection performance regarding the metal ions.

Heavy-metal-ion sensors are significant tools for environmental and food-analysis research because of the severe damage caused by toxic heavy metals to the organ systems of the human body^1^. Among the known toxic metals, lead and cadmium have received particular recognition due to their toxicity and related environmental pollution^2^. Low cost electrochemical sensors that can be operated easily have been developed for the detection of these and other heavy metal ions^3^,^4^. Further, the use of square wave anodic stripping voltammetry (SWASV) for heavy-metal-ion detection has gained popularity due to its effective selectivity and high sensitivity^5^,^6^.

In most cases, mercury-film electrodes are preferred for their excellent stripping characteristics in heavy metal ions recognition. However, the toxicity of mercury limits the applicability of these electrodes, especially in cases that involve water contact. Recently, the bismuth-film electrode was recognized as a promising substitute to the mercury electrode due to its low toxicity, large cathodic-potential range, and insensitivity to dissolved oxygen^7^,^8^. The sensitivity and low detection limit of bismuth-based electrochemical sensors are inadequate compared with those of conventional techniques such as atomic-absorption spectrophotometry, mass spectrometry, and inductively-coupled-plasma mass spectrometry^9^. Therefore, the development of a heavy-metal-ion sensor with high sensitivity is significant for point-of-care detection. Previously, a large amount of work has been undertaken regarding electrode-surface modifications to increase the sensitivity of metal-detection methods^10^,^11^,^12^,^13^. Among these works, the graphene-material-based working electrode has emerged as a promising alternative due to its unique thermal, mechanical, and electrochemical properties^14^.

Recently, reduced graphene oxide (RGO) has received increasing attention due to its applicability for the production of electronics, electrochemical, and biosensors^15^,^16^,^17^. Among different synthesis techniques for RGO, the solvothermal reduction of graphite oxide is the most attractive due to a simple setup for synthesis, sound scalability, and the ability to recover π-conjugation networks at a high temperature and pressure^18^,^19^. However, several problems arise from the solvothermal process. First, unreacted reducing agent remains on the RGO surface^20^. To resolve this issue, RGO gel was treated in acidic solution to remove the reduction agent on the RGO sheet^21^. Second, although RGO film directly patterned with a lithographic process is necessary for the development of high performing and miniaturized electrochemical sensors. A reliable and simple micro-fabrication process has not been developed yet because the solvent used for RGO suspension can be easily reacted with some negative or positive photoresists.

In this work, a simple and reliable micro-fabrication process is developed for the micro-patterning of TRGO film without affecting the electrochemical property of the film. An epoxy negative photoresist (SU-8) mold is applied for the lift off process of TRGO due to its good chemical resistance such as being highly resistant to solvents, acids, and corrosive environments^22^,^23^. After *in situ* deposition of the bismuth film on the working TRGO/Au electrode, a fully integrated heavy metal ions sensor with three electrodes is developed and characterized using SWASV. The fabricated sensor is small in size, cheap, and reliable in comparison with the existing and commercial sensors. It also provides quick response and full device integration on various substrates.

## Results

### Design and fabrication of the device

**Figure 1:**
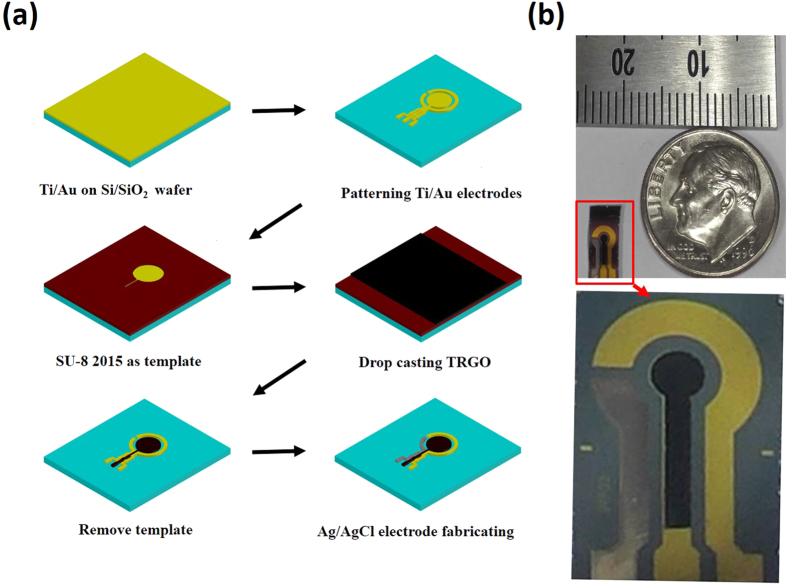
(**a**) Schematic illustration for the fabrication sequences of the micro-patterned TRGO film on the Au electrode and the proposed heavy-metal-detection sensor with three electrodes (working, counter, and reference electrodes); and (**b**) photomicrograph of fully integrated and miniaturized sensor.

The fabrication sequences of the heavy-metal-ion detection device on a silicon substrate are shown in Fig. 1(a). First, three different electrodes were patterned by using the wet-etching technique. Next, the TRGO was patterned in macro-scale on the top of the working electrode using the thick photoresist as a sacrificial layer. Finally, commercial Ag/AgCl paste was screen-printed on the top of the Au electrode surface as a reference electrode^24^. The gold paste sputtered on the substrate was used as the counter electrode. Photographs of the fabricated device are shown in Fig. 1(b).

### Physical characterization of the TRGO

**Figure 2:**
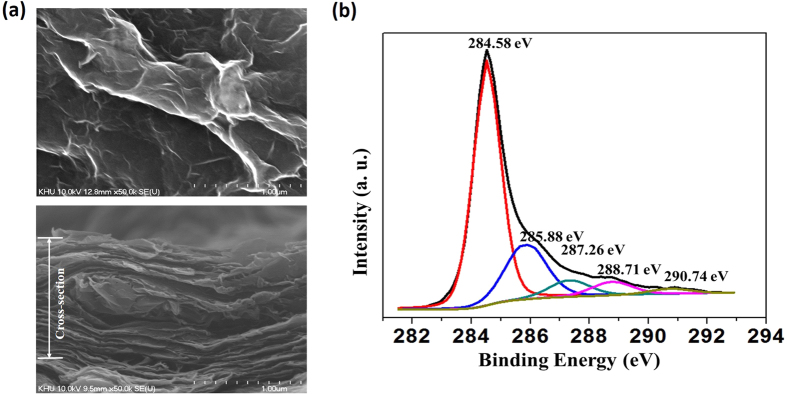
(**a**) Scanning-electron images showing the morphology of the TRGO/Au electrode (top and cross-section views); and (**b**) wide-range XPS spectra of the fabricated TRGO.

Figure 2(a) shows the surface morphology of the TRGO/Au electrode, whereby it is clearly observed that a rippled crumpling structure is on the TRGO sheets. This crumpling structure is supposedly caused by the reduction of the oxide groups in the RGO sheet.

An XPS analysis was carried out to determine the functional groups that are contained in the TRGO. The typical C1s spectra for the TRGO are shown in Fig. 2(b). Five different peaks indicate a considerable degree of oxidation, corresponding to the carbon atoms in the different functional groups. The peaks are centered at binding energies of 284.58 eV, 285.88 eV, 287.26 eV, 288.71 eV, and 290.74 eV. The peak at 284.58 eV corresponds to the non-oxygenated ring C involved in the C=C bonds for the production of sp^2^ hybridized carbon. Another peak (285.88 eV) is from the sp^3^ hybridized carbon and includes the C-C bond. The C in the C*-*O bonds (287.26 eV) includes hydroxyl and epoxy groups, the C in the C=O bonds (288.71 eV) includes carbonyl groups, and carbolic acids or ester groups are incorporated for the C in the O-C=O bonds (290.74 eV).

### Analytical performance of the sensor

**Figure 3:**
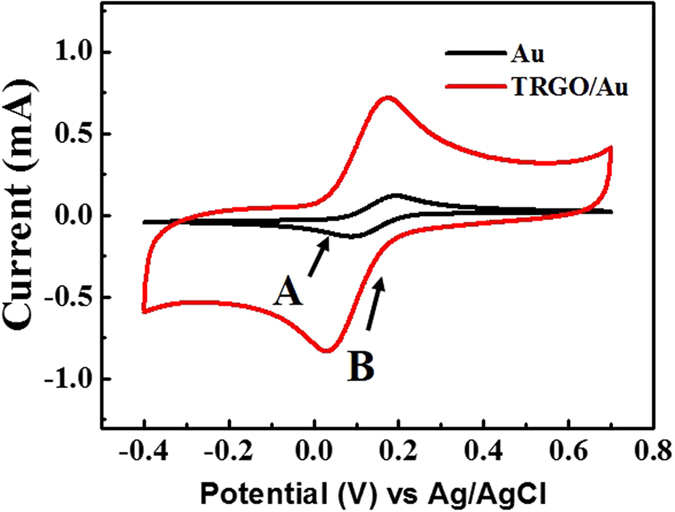
Cyclic voltammograms of TRGO/Au and Au electrodes in 0.1 M KCl containing 5 mM [Fe (CN)_6_]^3−/4−^ (Scan rate of 50 mV/s).

Cyclic voltammograms (scan rate = 50 mV/s) of the modified TRGO/Au electrode and gold electrode in a 0.1 M KCl solution containing 5 mM [Fe (CN)_6_]^3−/4−^ are shown in Fig. 3. On the Au electrode, a pair of weak redox peaks was observed. While on the TRGO/Au electrode, a pair of significantly-high peak currents was observed during the CV analysis. The performance of the fabricated sensor can be evaluated by the shapes and positions of the redox peaks in a 0.1 M KCl solution containing 5 mM [Fe (CN) _6_]^3−/4−^. Groups of CV curves were obtained by measuring the current of the TRGO working electrode using the fabricated electrode and a commercial reference electrode. The reference electrode was calibrated against commercial Ag/AgCl (3 M NaCl) electrode (Figure S4). In order to check the stability, we performed cyclic voltammetry over 10 cycles. Figure S5 shows the CV curves of the 1st and 10th cycle. The two CV curves are almost the same. This indicates that the as-fabricated sensor shows good stability. Figure S6 shows the CV curves of the fabricated sensor at different scan rates (25 mV/s, 50 mV/s, 75 mV/s, 100 mV/s). The shapes of all the CV curves are nearly identical and show clear redox peaks. The peaks show regular gradient slopes with increasing scan rates, and there is no obvious potential shift between the peaks. Figure S7 shows the CV curves of the TRGO working electrode before and after the patterning process. These curves are nearly the same, which indicates that the patterning process did not damage the electrochemical performance of the TRGO modified working electrode. In addition, no residues from the SU-8 photoresist were found on the TRGO layer, as clearly shown in the FESEM image in Figure S3.

**Figure 4:**
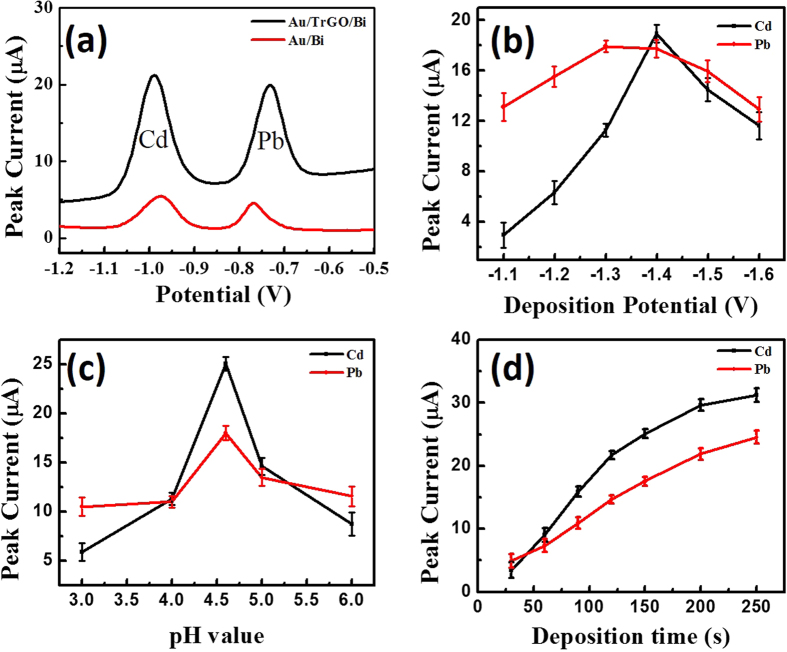
(**a**) Stripping voltammograms of Bi/Au and Bi/TRGO/Au in acetate-buffer solution containing 200 μg L^−1^ of Cd and Pb ions. Effects of the deposition potential for sensing target ions (**b**), pH value of the test solutions (**c**), and deposition time for sensing target ions (**d**) on the stripping-peak current of 200 μg L^−1^ of Cd and Pb ions. Data are presented with the mean of three replicates.

Figure 4(a) shows the SWASV of a 200 μg L^−1^ Cd-and-Pb-ion solution containing 600 μg of L^−1^ Bi ions on different electrodes. The striping peaks on the Bi/TRGO/Au-electrode-based sensor are higher than those on the Au/Bi-electrode-based sensor. This improved performance could be due to the presence of the TRGO, which offers abundant anchor sites for the deposition of heavy metal ions. The as-fabricated Bi/TRGO/Au electrochemical sensor was used for the detection of the Cd and Pb ions.

Figure 4(b) demonstrates the effect of deposition potentials that vary within a range of −1.1 V to −1.6 V on the stripping responses of the Cd and Pb ions at a deposition time of 150 s. When the deposition potential changed from −1.1 V to −1.6 V, the peak currents increased tremendously due to the more-complete reductions of the Cd and Pb ions. The highest stripping peak was observed when a −1.4 V deposition potential was used. With further negative shifting of the potential, the stripping response began to decrease gradually due to the enhancement of hydrogen evolution; furthermore, the hydrogen evolution was partially suppressed by the deposition of alloys on the electrode surface. A deposition potential of −1.4 V was therefore selected during the electrochemical analysis of the sensor.

The investigation results for the effect of pH on the determination of the Cd and Pb ions are shown in Fig. 4(c). The pH of the buffer solution has an extreme influence on the formation of the bismuth-metal alloy^25^. As the best signals appeared at pH 4.5, this pH level was used in subsequent experiments.

Figure 4(d) shows the influence of the stripping response on the deposition time within a range of 30 s to 250 s at a deposition potential of −1.4 V. The peak currents increased linearly with the extension of the deposition time. However, when the deposition time became longer than 150 s, the peak-current curve began to increase slightly in relation to the time, and this is probably due to the working electrode-surface saturation. Under the consideration of sensitivity, a determination time of 150 s was selected for the deposition of the ions.

**Figure 5:**
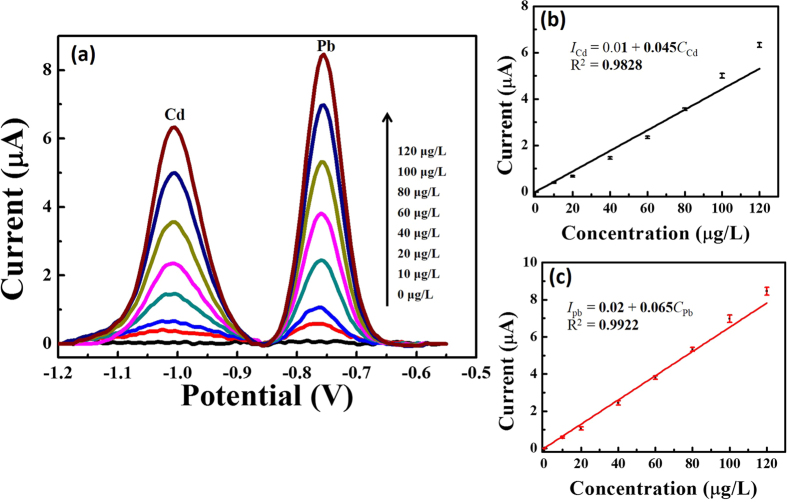
(**a**) Square-wave anodic-stripping voltammograms for different concentrations of Cd and Pb ions with the *in-situ*-plated Bi/TRGO/Au in 0.1 mol L^−1^ of acetate-buffer solution (pH 4.5) containing 600 μg L^−1^ of Bi ion. From bottom to top, 0 μg L^−1^, 10 μg L^−1^, 20 μg L^−1^, 40 μg L^−1^, 60 μg L^−1^, 80 μg L^−1^, 100 μg L^−1^, and 120 μg L^−1^. (**b,c**) Show the corresponding calibration curves of the Cd and Pb ions, respectively. Data are presented with the mean of three replicates.

Calibration curves for the simultaneous determination of the Cd and Pb ions were investigated under the optimal conditions in 0.1 M of the acetate-buffer solution. Figure 5(a) shows the stripping response of the as-fabricated device without any interference while the concentrations of the target metal ions were simultaneously increased from 0 μg L^−1^ to 120 μg L^−1^. The stripping-peak current and the concentrations of the Cd and Pb ions exhibited a favorable linear relationship. For the Cd ion (Fig. 5(b)), the linear-regression equation is calibrated as I_cd_ (current/μA) = 0.01 + 0.045C_Cd_ (concentration/μg L^−1^) (C_Cd_ = 0–120 μg L^−1^), with a correlation coefficient of 0.9828 (R^2^) and a sensitivity of 0.05 ± 0.01 μA μg^−1^L. For the Pb ion (Fig. 5(c)), the linear-regression equation is calibrated as I_pb_ (current/μA) = 0.02 + 0.065C_pb_ (concentration/μg L^−1^) (C_pb_ = 0–120 μg L^−1^), with a correlation coefficient of 0.9922 (R^2^) and sensitivity of 0.07 ± 0.01 μA μg^−1^L. Further, the fabricated micro-sensor also possesses satisfactory detection limits (S/N = 3) of 1.0 μg L^−1^ for the Cd ion and 0.4 μg L^−1^ for the Pb ion. The detection limits are lower than those of the other reported works, including the Bi Nps/SPCE detection limits of 5 μg L^−1^ for the Cd ion and 2 μg L^−1^ for the Pb ion^26^, the Bi/Au (with motor) detection limits of 0.7 μg L^−1^ for the Cd ion and 1.2 μg L^−1^ for the Pb ion^27^, and the Bi/ERGNO/SPE detection limits of 0.5 μg L^−1^ for the Cd ion and 0.8 μg L^−1^ for the Pb ion^28^.

**Figure 6:**
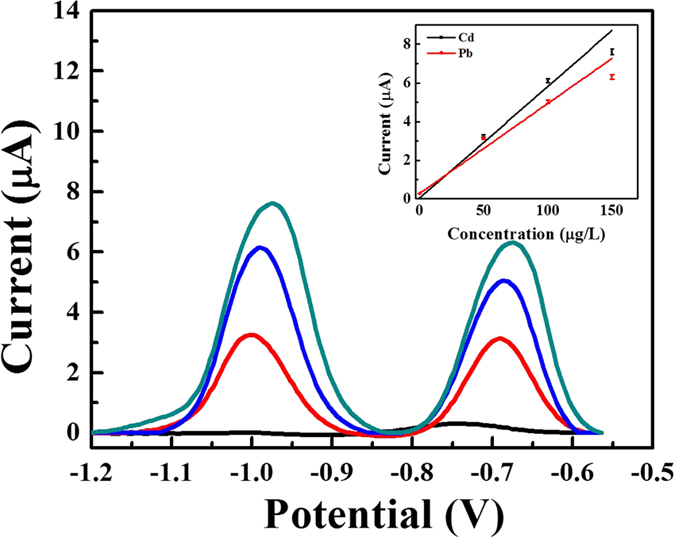
Typical stripping voltammograms for the detection of heavy metals in drinking water using the standard addition method under optimized conditions. The concentration of both Cd and Pb ions for each addition is 50 μg L^−1^. Inset: corresponding calibration curve. Data are presented with the mean of three replicates.

To evaluate the feasibility of the fabricated sensor for routine analysis, this sensor was applied to detect Cd and Pb ions in a drinking-water sample. Figure 6 illustrates the typical stripping voltammograms of the device when used for drinking-water-sample analysis. As shown, the stripping response for three standard additions exhibits good linearity, indicating the viability of the fabricated sensor in real sample analysis. The sensitivities for the Cd and Pb ions are 0.06 ± 0.01 μA μg^−1^L and 0.05 ± 0.01 μA μg^−1^L, respectively. The reproducibility of the fabricated micro-sensor was evaluated according to a repetitive measurement of 200 μg L^−1^ for both ions. The results show that our device is inexpensive and easily fabricated, while a wide range, low detection limit, and high sensitivity are also provided.

## Discussion

The sound film-forming ability of TRGO facilitates large surface coverage on the substrate, which is suitable for the large-scale production of TRGO. FESEM images (Figs S1 and S2) show that the TRGO covered all of the surfaces and is well patterned on the gold-coated substrate from the use of the developed technique. Figure S3 shows the FESEM image of the TRGO/Au surface after lithography. This figure clearly shows that there is no photoresist remaining on the TRGO surface.

According to the XPS-analysis data, the oxygenated peaks are much lower than the non-oxygenated peak. These results indicate that a high-quality RGO was developed through the use of the proposed techniques.

In Fig. 3, a pair of weak redox peaks can be observed on the Au electrode, whereas on the TRGO/Au electrode, a pair of significantly-high peak currents was found during CV analysis. These results indicate that reaction reversibility is possible in the developed microelectrode and the active surface area is enlarged. Clearly, the improved performance could be attributed to the unique nano-structure^29^. The CV peaks on the anodic curve are different for Au and TRGO/Au, while they are almost the same on the cathodic curve. This result may be due to the efficient electrocatalytic activity of TRGO, which facilitated the fast electron transfer kinetics^30^.

The electrochemical behaviors of the Pb and Cd heavy metal ions are different in different electrolytes such as HCl, HNO_3_, HClO_4_, N_2_SO_4_, acetate-buffer solution (NaAc-HAc), and phosphate-buffer solution (Na_2_HPO_4_-NaH_2_PO_4_). Among these electrolytes, the stripping performances of the Cd and Pb ions in 0.1 M of the acetate-buffer solution are the best due to the occurrence of well-defined peaks with the largest peak current^31^. To obtain the maximum sensitivity for the detection of heavy metal ions, this work investigated the effects of different parameters in 0.1 M of an acetate-buffer solution containing 200 μg L^−1^ of the Cd and Pb ions.

The fabricated micro-sensor shows correlation coefficients of 0.9828 and 0.9922 for the Cd (Fig. 4(b)) and Pb (Fig. 4(c)) ions, respectively; moreover, the fabricated micro-sensor exhibited satisfactory detection limits of 1.0 μg L^−1^ and 0.4 μg L^−1^ for the Cd and Pb ions, respectively. Lower detection limits could be achieved for both of the target metal ions by prolonging the detection time. Drinking-water samples were used to test the suitability of the proposed electrochemical sensor for real-life applications. The sensor exhibited acceptable performance in terms of the detection of both Cd and Pb ions in drinking water.

In conclusion, a working Bi/TRGO/Au-modified electrode based on a fully integrated electrochemical sensor was developed and subsequently used for a simultaneous SWASV determination of Cd and Pb ions. The developed sensor exhibited a high and sharp peak current for the target heavy metal ions due to the outstanding properties of the TRGO film and the sound stripping characteristics of the bismuth film. Due to the unique properties of TRGO, the fabricated device exhibited sound sensitivity and high stability with simple and green preparation methods.

## Methods

### Materials and apparatus

The standard solutions of all of the metals (Cd ion, Pb ion, Bi ion), the graphite powder (44-μm size), the β-D (+) glucose, the N, N-dimethylformamide (DMF), the sodium acetate, Ag/AgCl paste (ALS Co, Japan), and the glacial acetic acid were purchased from Aldrich Co. (St. Louis, USA). Acetate-buffer solution (0.1 M) served as the supporting electrolyte for the detection of the heavy metals. Deionized water (resistivity ≥ 18 MΩ.cm) was used for all of the experiments and all of the electrochemical measurements were carried out in a 20 mL cell.

An electrochemical analyzer (Model 660D series, CH Instruments Inc., USA) was used for the electrochemical experiments on the fabricated electrode at room temperature. A three-electrode system, including a fabricated electrode that functioned as a working electrode, was used, while an Ag/AgCl electrode with 3 M of NaCl and a flat Pt-bar electrode were utilized as the reference and counter electrodes, respectively. The physical characteristics of the TRGO and the sensor electrodes were investigated using high-resolution XPS and field emission scanning electron microscopy (FESEM).

### Synthesis of the TRGO

The graphite oxide was prepared using a modified Hummer’s method^32^. TRGO was synthesized using a modified hydrothermal reduction technique^21^,^33^. Briefly, 52 mg of the graphite oxide was added to 26 mL of deionized water, followed by ultrasonication for approximately 30 min. The as-prepared mixture solution and 0.1 M of glucose were then mixed and the resultant mixture was allowed to stand for 1 h. Next, the mixture was sealed in a teflon-lined autoclave and maintained at 180 °C for 2 h in a convection oven. When the autoclave had cooled, the as-prepared gel was dispersed again in 1 M of an acetic-acid aqueous solution, after which time it was left for 5 h. Finally, the mixture was washed with doubly-distilled water until a pH of 7 was reached. It was then dried overnight in an oven at 90 °C under a vacuum.

### Fabrication of micro-patterned TRGO/Au-based electrochemical sensor

Figure 1(a) shows the fabrication sequence of the proposed micro-sensor. An insulation layer was first deposited on top of the silicon substrate. After a Cr and Au film was sputtered on top of the SiO_2_ layer, it was patterned with three different electrodes using the wet-etching technique. Next, the photolithography technique was used to cover the sample with an SU-8 photoresist, whereby the working electrode was excluded. Then, following drop-casting of 50 μl of the TRGO suspension, the working electrode was dried in the air at room temperature for 2 h. Next, the photoresist was removed from the sample using a PG-remover, and washed away with IPA and deionized water. Finally, Ag/AgCl paste was screen-printed on top of the reference Au electrode and dried at 120 °C for 5 min. Figure 2(b) shows a photograph of the fabricated heavy-metal-ion micro-sensor with the working TRGO/Au electrode.

### Measurement procedure

The micro-sensor was applied using an *in situ* electroplating Bi, whereby the fabricated device was immersed in 20 mL of a metal-ion standard solution. The SWASV analyses of the Cd and Pb ions were performed in 0.1 M acetate-buffer solutions in the presence of 600 μg L^−1^ of Bi^3+^. The sensing principle for the heavy metal ions using SWASV is shown in Figure S8. The SWASV mode contains a time-controlled electrochemical deposition (Figure S8(a)) and a positively-applied-potential square-wave stripping scan (Figure S8(b)). The parameters are as follows: E_dep_ = 150 s; t_eq_ = 20 s; E_begin_ = −1.4 V, E_end_ = −0.4 V, E_step_ = 5 mV, E_ampl_ = 25 mV, and ƒ = 25 Hz. Prior to the next cycle, a 60 s clean step at 0.1 V was performed to remove the residual bismuth. A magnetic stirrer was used to stir the test solutions during the electrodeposition and cleaning steps.

## Additional Information

**How to cite this article**: Xuan, X. *et al*. A Fully Integrated and Miniaturized Heavy-metal-detection Sensor Based on Micro-patterned Reduced Graphene Oxide. *Sci. Rep.* **6**, 33125; doi: 10.1038/srep33125 (2016).

## Supplementary Material

Supplementary Information

## References

[b1] Aragay G. & Merkoçi A. Nanomaterials application in electrochemical detection of heavy metals, Electrochim. Acta. 84, 49–61 (2012).

[b2] Kemper T. & Sommer S. Estimate of heavy metal contamination in soils after a mining accident using reflectance spectroscopy. Environ. Sci. Technol. 36, 2742–2747 (2002). 12099473 10.1021/es015747j

[b3] Lee G. J., Kim C. K., Lee M. K. & Rhee C. K. Simultaneous Voltammetric Determination of Zn, Cd and Pb at Bismuth Nanopowder Electrodes with Various Particle Size Distributions. Electroanalysis 22, 530–535 (2010).

[b4] Ouyang R. . Simultaneous Stripping Detection of Zn(II), Cd(II) and Pb(II) Using a Bimetallic Hg-Bi/single-walled Carbon Nanotubes Composite Electrode. J. Electroanal. Chem. 656, 78–84 (2011). 10.1016/j.jelechem.2011.01.006 PMC3108327 21660117

[b5] Jothimuthu P. . Lab-on-a-chip Sensor for Detection of Highly Electronegative Heavy Metals by Anodic Stripping Voltammetry. Biomed. Microdevices. 13, 695–703 (2011). 21479538 10.1007/s10544-011-9539-1 PMC3824972

[b6] Krolicka A. . Study on Catalytic Adsorptive Stripping Voltammetry of Trace Cobalt at Bismuth Film Electrodes. Electroanalysis 15, 1859–1863 (2003).

[b7] Lee G. J., Lee H. M. & Rhee C. K. Bismuth Nano-powder Electrode for Trace Analysis of Heavy Metals Using Anodic Stripping Voltammetry. Electrochem. Commun. 9, 2514–2518 (2007).

[b8] Economou A. Bismuth-film Electrodes: Recent Developments and Potentialities for Electroanalysis. Trends Anal. Chem. 24, 334–340 (2005).

[b9] Injang U. . Determination of Trace Heavy Metals in Herbs by Sequential Injection Analysis-anodic Stripping Voltammetry Using Screen-printed Carbon Nanotubes Electrodes. Anal. Chim. Acta. 668, 54–60 (2010). 20457302 10.1016/j.aca.2010.01.018

[b10] Veronika U., Martin B., Karel V. & Alexander K. Porous Bismuth Film Electrode for Signal Increase in Anodic Stripping Voltammetry. Electroanalysis 22, 1524–1530 (2010).

[b11] Sahoo P. K. . *In Situ* Synthesis and Properties of Reduced Graphene Oxide/Bi Nanocomposites: As an Electroactive Material for Analysis of Heavy Metals. Biosensors and Bioelectronics 43, 293–296 (2012). 23334218 10.1016/j.bios.2012.12.031

[b12] Wang Z., Liu G., Zhang L. & Wang H. A Bismuth Modified Hybrid Binder Carbon Paste Electrode for Electrochemical Stripping Detection of Trace Heavy Metals in Soil. Int. J. Electrochem. Sci. 7, 12326–12339 (2012).

[b13] Gregory M., Tuan D. N. & Benoit P. Modified Electrodes Used for Electrochemical Detection of Metal Ions in Environmental Analysis. Biosensors. 5, 241–275 (2015). 25938789 10.3390/bios5020241 PMC4493548

[b14] Shao Y. Y. . Graphene Based Electrochemical Sensors and Biosensors: A Review. Electroanalysis 20, 1027–1036 (2010).

[b15] Robinson J. T. . Reduced Graphene Oxide Molecular Sensors. Nano Lett. 8, 3137–3140 (2008). 18763832 10.1021/nl8013007

[b16] Wang T. . A Review on Graphene-Based Gas/Vapor Sensors with Unique Properties and Potential Applications. Nano-Micro Lett. 8, 95–119 (2016). 10.1007/s40820-015-0073-1 PMC6223682 30460270

[b17] Service R. F. Carbon Sheets an Atom Thick Give Rise to Graphene Dreams. Science 324, 875 (2009). 19443761 10.1126/science.324_875

[b18] Dubin S. . A One-Step, Solvothermal Reduction Method for Producing Reduced Graphene Oxide Dispersions in Organic Solvents. ACS Nano 4, 3845–3852 (2010). 20586422 10.1021/nn100511a PMC3939021

[b19] Pham V. H. . Chemical functionalization of graphene sheets by solvothermal reduction of a graphene oxide suspension in N-methyl-2-pyrrolidone. J. Mater. Chem. 21, 3371–3377 (2011).

[b20] Wang H., Robinson J. T., Li X. & Dai H. Ni (OH)_2_ Nanoplates Grown on Graphene as Advanced Electrochemical Pseudocapacitor Materials. J. Am. Chem. Soc. 131, 9910–9911 (2009). 20443559 10.1021/ja102267j

[b21] Hossain M. F. & Park J. Y. Plain to Point Network Reduced Graphene Oxide – Activated Carbon Composites Decorated with Platinum Nanoparticles for Urine Glucose Detection. Sci. Reports. 6, 1–10 (2016). 10.1038/srep21009 PMC4753453 26876368

[b22] Lee J. S. & Lee S. S. Fabrication of a freestanding micro mechanical structure using electroplated thick metal with a HAR SU-8 mold. Microsyst Technol. 15, 287–296 (2009).

[b23] Carlos J. H. & Thomas G. M. Colloidal Alphabet Soup: Monodisperse Dispersion of Shape-Designed Lithoparticles. J. Phys. Chem. C. 111, 4477–4480 (2007).

[b24] Akhtar H. & Jean L. M. Disposable Screen Printed Electrochemical Sensors: Tools for Enviromental Monitoring. Sensors 14, 10432–10453 (2014). 24932865 10.3390/s140610432 PMC4118360

[b25] Wang J. Stripping analysis at bismuth electrodes. Electroanalysis 17, 15–16 (2005).

[b26] Cadevall M., Ros J. & Merkoc A. Bismuth nanoparticles integrationinto heavy metal electrochemical strippingsensor. Electrophoresis 36, 1872–1879 (2015). 25994368 10.1002/elps.201400609

[b27] Zhang W., Zhang H., Williams S. E. & Zhou A. H. Microfabricated three-electrode on-chip PDMS device with a vibration motor for stripping voltammetric detection of heavy metal ions. Talanta 132, 321–326 (2015). 25476314 10.1016/j.talanta.2014.08.075

[b28] Ping J., Wang Y., Wu J. & Ying Y. Development of an electrochemically reduced graphene oxide modified disposable bismuth film electrode and its application for stripping analysis of heavy metals in milk. Food Chemistry 151, 65–71 (2013). 24423503 10.1016/j.foodchem.2013.11.026

[b29] Wang Z., Wang H., Zhang Z. & Liu G. Electrochemical determination of lead and cadmium in rice by a disposable bismuth/electrochemically reduced graphene/ionic liquid composite modified screen-printed electrode. Sens. Actuators, B. 199, 7–14 (2014).

[b30] Chen L. . Direct electrodepositon of reduced graphene oxide on glassy carbon electrode and its electrochemical application. Electrochemistry Communications 13, 133–137 (2011).

[b31] Pei S. & Cheng M. H. The reduction of graphene oxide. Carbon 50, 3210–3228 (2012).

[b32] Xu Y., Bai H., Lu G., Li C. & Shi G. Flexible Graphene Films via the Filtration of Water-Soluble Noncovalent Functionalized Graphene Sheets. J. Am. Chem. Soc. 130, 5856–5857 (2008). 18399634 10.1021/ja800745y

[b33] Xu Y. . Flexible Solid-State Supercapacitors Based on Three-Dimensional Graphene Hydrogel Films. Acs Nano 7, 4042–4049 (2013). 23550832 10.1021/nn4000836

