# Supplementary material for: A Fully Integrated and Miniaturized Heavy-metal-detection Sensor Based on Micro-patterned Reduced Graphene Oxide
Source: Sci Rep. 2016 Sep 12;6:33125. doi: 10.1038/srep33125 (PMC5018876; doi:10.1038/srep33125)
Supplement: Supplementary Information [file srep33125-s1.pdf]

A Fully Integrated and Miniaturized Heavy-metal-detection Sensor Based on Micro-patterned Reduced Graphene Oxide

Xing Xuan, Md. Faruk Hossain, Jae Yeong Park*

Supplementary Data

Department of Electronic Engineering, Micro/Nano Devices & Packaging Lab., Kwangwoon University, 447-1, Wolgye-Dong, Nowon Gu, Seoul, 139-701, Korea

* Corresponding author. Tel.: +82-2-940-5113; fax: +82-2-942-1502;
e-mail: jaepark@kw.ac.kr

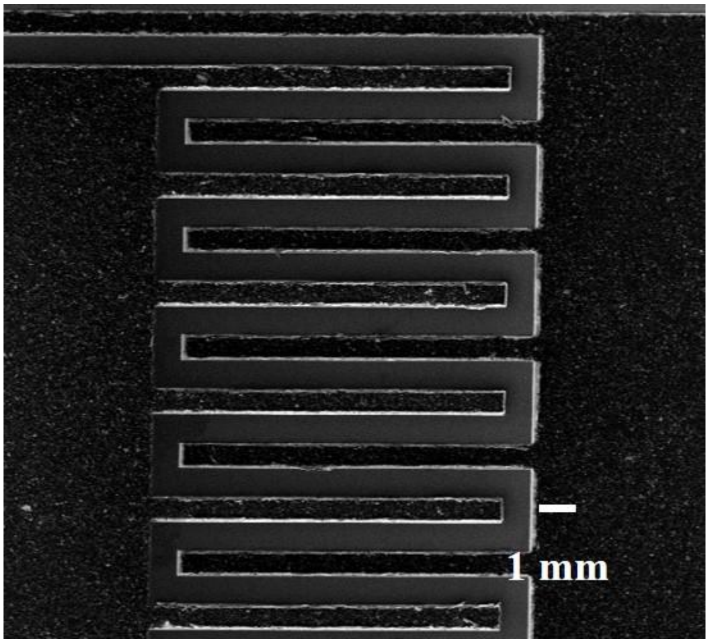

Figure S1. Top view of FE-SEM image of micro-patterned TRGO with an interdigital shape.

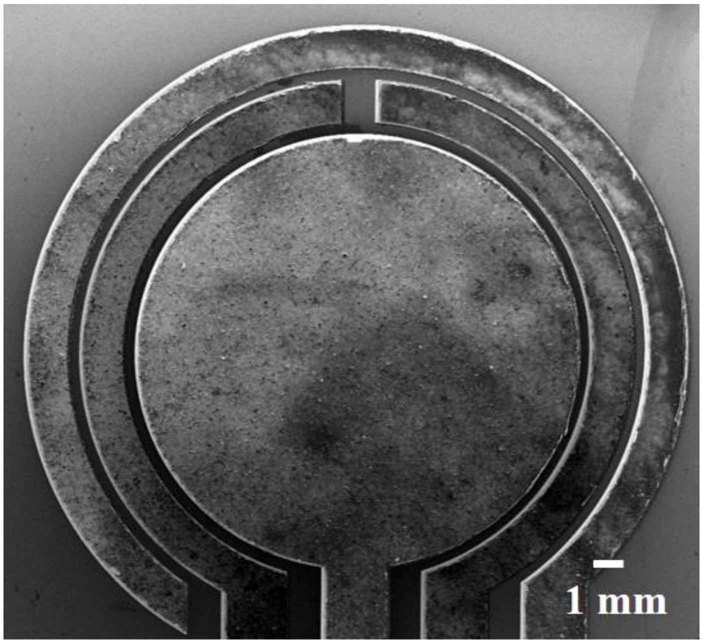

Figure S2. Top view of FE-SEM image of micro-patterned TRGO with a disk shape.

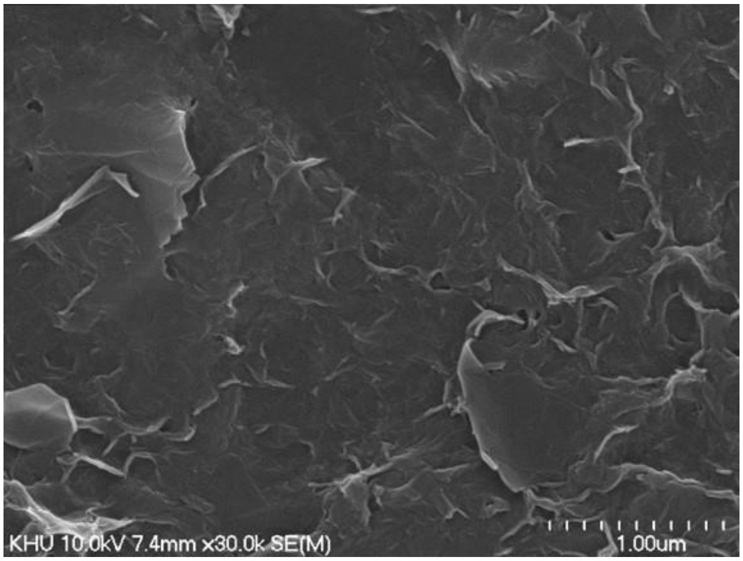

Figure S3. The FE-SEM image of the after patterning TRGO/Au electrode.

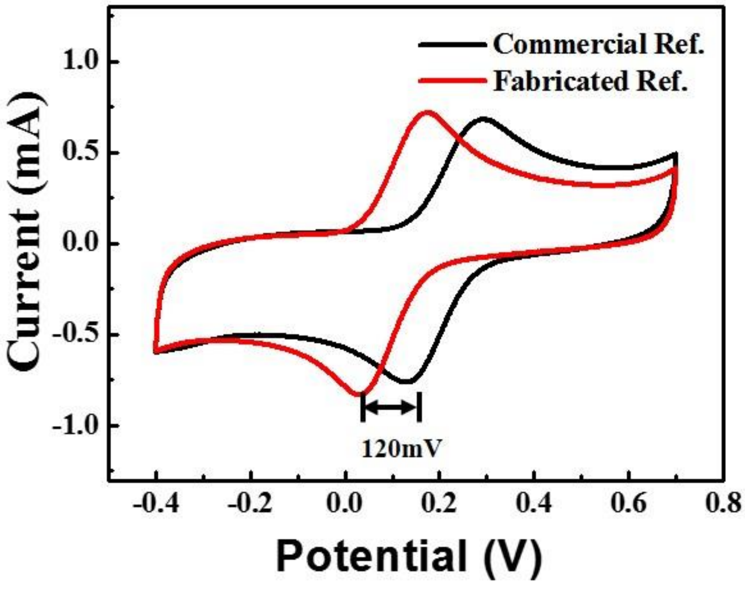

Figure S4. CV curves of fabricated reference electrode and commercial Ag/AgCl (3M NaCl) electrode in 0.1 M KCl solution containing 5 mM $[Fe(CN)_6]^{3-/4-}$.

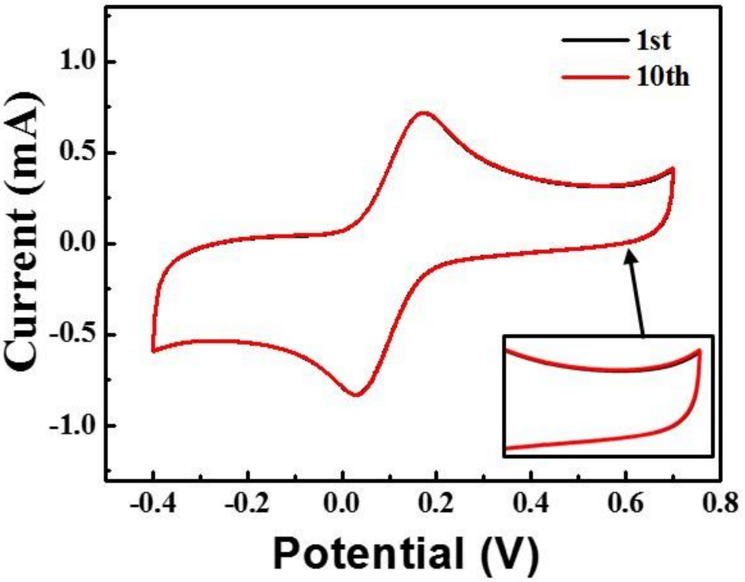

Figure S5. The stability of fabricated sensor with screen printed reference electrode.

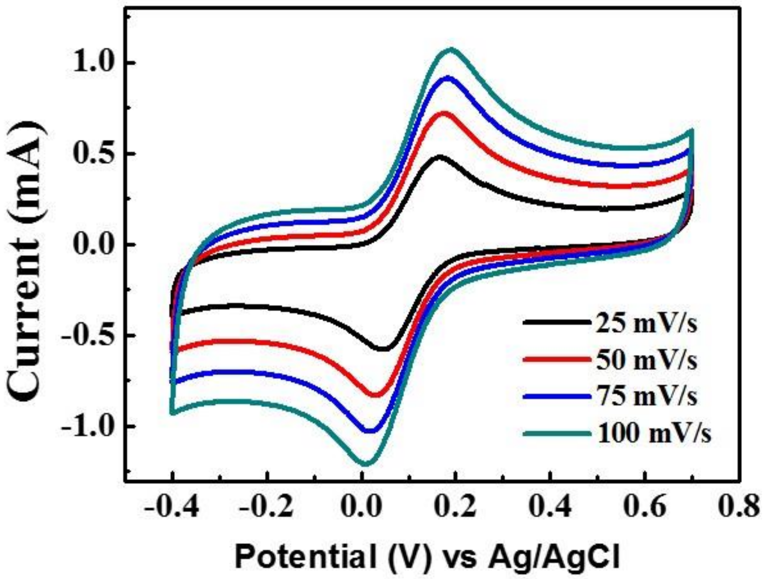

Figure S6. The CV curves of working electrode in 0.1 M KCl containing 5 mM $[Fe\ (CN)_6]^{3-/4-}$ at 25, 50, 75 and 100 mV/s scan rates .

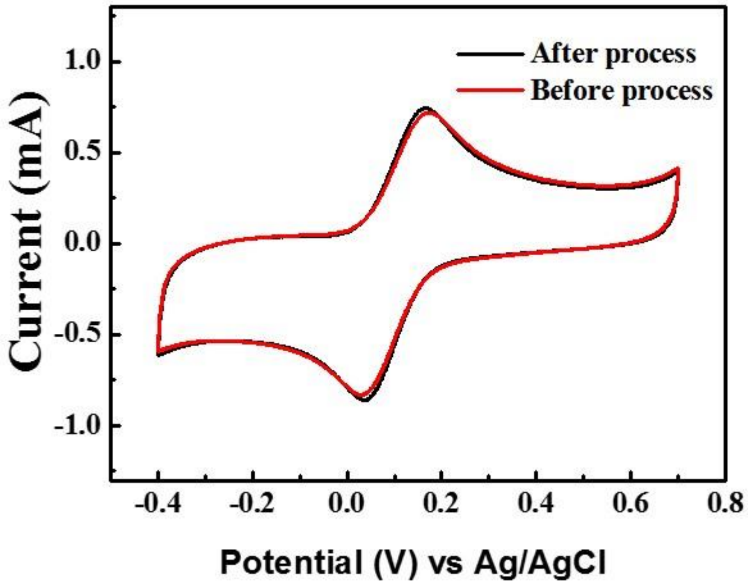

Figure S7. The TRGO/Au electrodes CV curves for solution containing 5 mM [Fe (CN)$_6$]$^{3-/4-}$ in 0.1 M KCl, (red line) before and (black line) after patterning process (Scan rate of 50 mV/s).

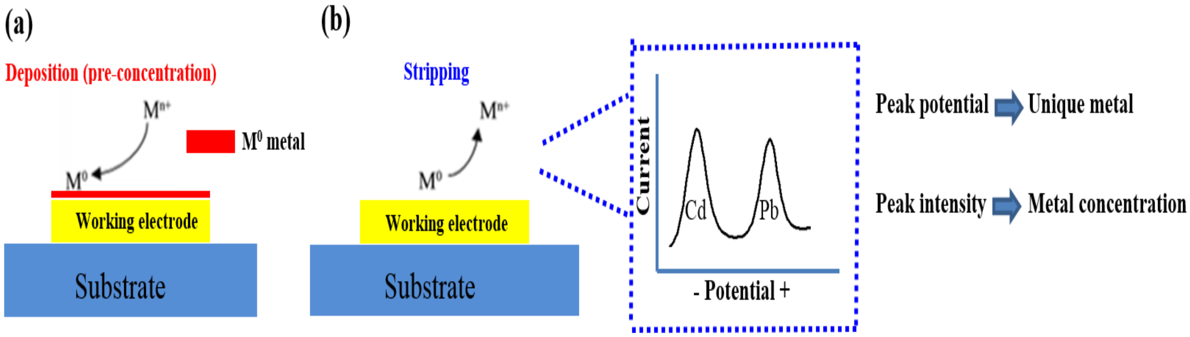

Figure S8. The heavy metal ions sensing principle using SWASV.
